# Functional, Radiological, and Neuromuscular Outcomes Following Reverse-Passing Anterior Cruciate Ligament Reconstruction With Anterolateral Ligament Reconstruction Using Hamstring Autograft

**DOI:** 10.7759/cureus.113717

**Published:** 2026-07-31

**Authors:** Simone Giusti, Rosaria Merlicco, Vittorio Alfonsi, Ezio Adriani

**Affiliations:** 1 Trauma and Orthopedics, Fondazione Policlinico Universitario A. Gemelli, Istituto di Ricovero e Cura a Carattere Scientifico (IRCCS), Rome, ITA; 2 Rehabilitation, Fondazione Policlinico Universitario A. Gemelli, Istituto di Ricovero e Cura a Carattere Scientifico (IRCCS), Rome, ITA

**Keywords:** anterior cruciate ligament, anterolateral ligament, graft maturation, hamstring autograft, return to sport

## Abstract

Introduction: Combined anterior cruciate ligament (ACL) and anterolateral ligament (ALL) reconstruction has gained increasing attention for improving rotational stability and reducing graft failure rates, but early outcomes following novel techniques such as the reverse-passing reconstruction technique remain poorly characterized. Materials and methods: Sixty-four consecutive patients underwent primary ACL reconstruction using a single-surgeon reverse-passing hamstring autograft technique with simultaneous ALL reconstruction. Functional outcomes (Knee Injury and Osteoarthritis Outcome Score (KOOS), International Knee Documentation Committee Subjective Knee Evaluation Form (IKDC), and Lysholm Knee Scoring Scale), MRI-based graft maturation (signal-to-noise quotient (SNQ)), and quadriceps and hamstring limb symmetry indices (LSI) were assessed at six months. Activity level (Tegner) and return-to-sport status were assessed at 12 months. Correlation and multivariable regression analyses examined relationships between SNQ and functional and strength outcomes. Results: At six months, mean KOOS was 88.9 ± 8.7, IKDC 86.8 ± 6.7, and Lysholm 88.6 ± 6.6. The mean SNQ was 4.86 ± 1.55 and correlated strongly with quadriceps LSI (r = −0.87, P < 0.001) and hamstring LSI (r = −0.95, P < 0.001), but not with patient-reported outcomes. Multivariable regression confirmed SNQ as an independent predictor of quadriceps strength recovery but not subjective function. Tegner scores returned to pre-injury levels by 12 months (P = 0.544), and all patients (100%) had returned to sport by 12 months. No graft failures, infections, or neurovascular complications occurred; one patient (1.6%) required surgical revision for hardware-related wound dehiscence overlying Gerdy's tubercle. Conclusions: In this single-arm preliminary series, reverse-passing ACL and ALL reconstruction were associated with excellent six-month functional recovery, a strong correlation between graft maturation and neuromuscular but not subjective recovery, a high rate of return to sport by 12 months, and a low complication rate. Comparative studies with a control group are needed to determine whether these outcomes are attributable to the reverse-passing technique itself.

## Introduction

Anterior cruciate ligament (ACL) rupture remains one of the most frequent injuries in young and active populations, particularly among athletes involved in pivoting and contact sports. Although ACL reconstruction reliably restores anterior tibial translation, residual rotational laxity, persistent instability, and graft failure remain important clinical challenges, especially in younger and high-risk patients [1-2].

Recently, increasing attention has been directed toward the anterolateral structures of the knee, including the anterolateral ligament (ALL) and other components of the anterolateral complex. Biomechanical studies have shown that these structures contribute substantially to the control of tibial internal rotation and the pivot-shift phenomenon [3-4]. As a result, lateral extra-articular procedures such as ALL reconstruction and lateral extra-articular tenodesis (LET) have been increasingly combined with ACL reconstruction to improve rotational control and reduce graft overload [5-6].

Several studies have demonstrated the benefits of adding a lateral extra-articular procedure to ACL reconstruction. A recent study reported a significantly lower graft rupture rate in elite alpine skiers undergoing ACL reconstruction combined with LET compared with isolated ACL reconstruction [1]. Similarly, another team found that LET reduced revision rates in elite athletes [7] and demonstrated improved clinical outcomes and lower residual instability in adolescent patients undergoing combined ACL and LET reconstruction [8]. In the STABILITY study, the investigators identified younger age, higher activity level, and greater rotational laxity as major predictors of graft failure, while demonstrating that adding LET reduced the risk of graft rupture in young active individuals [9].

Recent systematic reviews and meta-analyses have further reinforced these findings, demonstrating that the addition of a lateral extra-articular procedure significantly reduces graft failure rates compared with isolated ACL reconstruction at a minimum follow-up of two years [2]. Similar conclusions [6,10-11] have also been reported by current reviews, all of whom found that combined procedures improve rotational stability, reduce residual laxity, and may decrease revision rates without substantially increasing complications [11].

As evidence supporting combined reconstruction continues to grow, efforts have also focused on identifying which patients may benefit most from these procedures. Recent international consensus statements and systematic reviews have identified several indications for adding a lateral extra-articular procedure, including young age, participation in pivoting sports, generalized ligamentous laxity, high-grade pivot shift, revision surgery, meniscal deficiency, and elite athletic status [12-13].

In addition to improvements in rotational stability, there has been increasing interest in understanding the biological processes underlying graft healing and maturation following ACL reconstruction. MRI has become one of the most widely used tools to assess graft remodeling, with the signal-to-noise quotient (SNQ) representing a surrogate marker of graft ligamentization. However, previous studies have shown inconsistent relationships between MRI-derived graft signal and clinical recovery, particularly regarding subjective functional scores.

Technical refinements in ACL reconstruction have also focused on improving graft fixation and biological integration. Biomechanical studies have suggested that fixation methods play a major role in graft incorporation, with interference screw fixation providing high initial stability [14]. Numerous papers have described the technical rationale for combining ACL reconstruction with LET [5], with a few studies suggesting that ALL reconstruction and LET provide comparable biomechanical improvements in rotational stability [4].

Despite the increasing popularity of combined ACL and ALL reconstruction, limited evidence is available regarding the early clinical, radiological, and neuromuscular outcomes of newer fixation strategies such as the reverse-passing technique. The reverse-passing method was developed to address specific biomechanical limitations of conventional ACL reconstruction, including suboptimal graft tensioning at the tibia (due to the lack of direct visualization) and the need to preserve sufficient autograft length for simultaneous ALL reconstruction using the same autograft. These advantages are described in detail in the technique paper cited herein [15]. The primary aim of the present study was therefore to report early functional, radiological, and neuromuscular outcomes following reverse-passing ACL reconstruction with concomitant ALL reconstruction. Secondary aims were (i) to explore the association between MRI-derived graft maturation and objective neuromuscular recovery; (ii) to examine whether graft maturation correlated with patient-reported functional outcomes; and (iii) to assess activity level and return-to-sport status at 12 months. This study is not intended to demonstrate the superiority of the reverse-passing technique over conventional ACL reconstruction, which would require a comparative study design, but rather to provide preliminary outcome data to inform future controlled investigations.

## Materials and methods

This study was approved by the Institutional Ethics Committee of Università Cattolica del Sacro Cuore on January 22, 2026. Given the retrospective observational nature of the study, involving only the analysis of routinely collected clinical data with no additional interventions or patient contact, formal IRB review beyond institutional notification was not required. All patients provided written informed consent for the use of their clinical data for research purposes at the time of their original clinical assessment. A retrospective cohort study at the Complex Operational Unit of Sports Traumatology and Joint Reconstruction was conducted between January 2023 and January 2025, corresponding to Level of Evidence IV. Data were collected retrospectively from the institutional electronic medical records and operative database, including preoperative clinical notes, intraoperative surgical records, postoperative follow-up assessments, and MRI and radiographic reports. A total of 64 consecutive patients who had undergone primary ACL reconstruction with the reverse-passing technique were included. Inclusion criteria consisted of patients aged ≥16 years with a confirmed ACL rupture (clinical and MRI) who had completed a full 12-month follow-up period. Exclusion criteria included revision surgery, multiligament injuries, and incomplete follow-up.

Surgical technique

All procedures were performed by a single senior orthopedic surgeon, fellowship-trained in sports medicine (E.A.). All patients received the same ACL and ALL reconstruction as described by the reverse-passing technique [15]: a retro-drilled tibial half-tunnel and an outside-in femoral tunnel were created; the graft was threaded proximally and distally before being fixed at the femoral end with an interference screw, while tibial fixation was achieved through a suspension system. The remaining graft was used to reinforce the ALL, passing deep to the lateral collateral ligament and iliotibial band, and was anchored to Gerdy's tubercle via a knotless system.

Postoperative rehabilitation and outcome assessment

Postoperative rehabilitation followed a standardized protocol emphasizing early mobilization, progressive loading, and neuromuscular training with proprioceptive focus. Patients were evaluated at six months postoperatively using the Knee Injury and Osteoarthritis Outcome Score (KOOS) [16], a five-subscale patient-reported outcome measure (pain, symptoms, activities of daily living, sports and recreation, and quality of life) scored from 0 to 100, where higher scores indicate better knee health; the International Knee Documentation Committee Subjective Knee Evaluation Form (IKDC) [17], an 18-item questionnaire scored from 0 to 100, where higher scores reflect less symptom burden and better function; and the Lysholm Knee Scoring Scale [18], an eight-item scale scored from 0 to 100 (scores ≥95: excellent; 84-94: good; 65-83: fair; <65: poor), and at 12 months using the Tegner activity scale [19], an 11-point ordinal scale (0 = on sick leave or disability pension to 10 = participation in national or international elite competitive sports), which classifies activity level and allows comparison with pre-injury scores. MRI was used to assess graft maturation via the SNQ. Isokinetic testing assessed quadriceps and hamstring strength using a Biodex System 4 isokinetic dynamometer (Biodex Medical Systems, Shirley, NY) at 60°/s. The limb symmetry index (LSI) was calculated as $\frac{\text{Involved limb peak torque}}{\text{Uninvolved limb peak torque}} \times 100$, expressed as a percentage; values ≥90% are generally considered indicative of adequate symmetry for return-to-sport clearance. The hamstring-to-quadriceps (H/Q) ratio was calculated as $\frac{\text{Hamstring peak torque}}{\text{Quadriceps peak torque}} \times 100$, expressed as a percentage; a conventional H/Q ratio ≥60% is considered the threshold for adequate knee stability and neuromuscular balance.

MRI was performed using a 3-T scanner with sagittal proton-density-weighted, fat-suppressed sequences. The SNQ was calculated as $\frac{\mathrm{SI}_{\mathrm{graft}} - \mathrm{SI}_{\mathrm{background}}} {\mathrm{SI}_{\text{reference tissue}} - \mathrm{SI}_{\mathrm{background}}}$, where SI (signal intensity) is the mean MRI signal value measured in a defined region of interest; SI graft was measured in the midsubstance of the intra-articular graft on the sagittal oblique image best depicting its long axis, and SI reference was measured in the posterior cruciate ligament, with SI background sampled from an artifact-free region outside the knee. All measurements were performed independently by 2 blinded observers, and interobserver reliability was assessed using intraclass correlation coefficients (ICC = 0.91; 95% CI, 0.85-0.95).

Statistical analysis

All statistical analyses were performed using SPSS Statistics version 29.0 (IBM Corp. Released 2022. IBM SPSS Statistics for Windows, Version 29.0. Armonk, NY). Continuous variables were assessed for normality using the Shapiro-Wilk test and inspection of Q-Q plots. As all variables demonstrated a normal distribution, they were reported as mean ± standard deviation. Paired comparisons between pre-injury and postoperative Tegner scores were conducted using a paired Student's t-test. Effect sizes were calculated using Cohen's d. Correlation analyses were performed using Pearson correlation coefficients to assess relationships between SNQ values and both functional and strength outcomes. Multivariable regression analysis was conducted to evaluate whether demographic variables and SNQ independently predicted functional outcomes. Statistical significance was set at P < 0.05.

## Results

A total of 64 patients completed the 12-month follow-up and were included in the final analysis. The study cohort consisted of 41 females and 23 males, with a mean age of 22.2 ± 6.5 years and a mean body mass index of 22.3 ± 2.3 kg/m². The relatively high proportion of female patients in this cohort is notable, as ACL reconstruction series more commonly report male predominance; this may reflect referral patterns or sport participation trends specific to our practice and warrants further investigation in future studies. No intraoperative complications, graft failures, or infections were observed during the study period. One patient (1.6%) required surgical revision of the wound overlying Gerdy's tubercle for hardware-related wound dehiscence at four months postoperatively, classified as a Clavien-Dindo grade IIIb complication. Patient demographics are summarized in Table 1.

**Table 1:** Demographic characteristics (n = 64) Values are presented as mean ± SD or number (%). BMI: body mass index, SD: standard deviation

Variable	Value
Age at surgery (y)	22.2 ± 6.5
BMI (kg/m²)	22.3 ± 2.3
Female sex	41 (63.6)
Male sex	23 (36.4)
Follow-up duration (mo)	12

At six months postoperatively, patients demonstrated high levels of functional recovery. The mean KOOS score was 88.9 ± 8.7, while the mean IKDC subjective score and Lysholm score were 86.8 ± 6.7 and 88.6 ± 6.6, respectively, indicating favorable early postoperative knee function following reconstruction. No statistically significant differences in functional outcomes were observed between male and female patients (Table 2; P > 0.05 for all comparisons); this sex-based comparison was exploratory and was not a primary aim of the study.

**Table 2:** Functional outcome scores at six months by sex Values are mean ± SD. Between-sex comparisons performed using independent-samples Welch's t-test. t-value, Welch's t statistic; Cohen's d, effect size. KOOS (0-100, higher = better); IKDC (0-100, higher = better); and Lysholm (0-100; ≥95 excellent, 84-94 good, 65-83 fair, <65 poor). SD: standard deviation, KOOS: Knee Injury and Osteoarthritis Outcome Score, IKDC: International Knee Documentation Committee Subjective Knee Evaluation Form, Lysholm: Lysholm Knee Scoring Scale

Outcome	Male (n = 23)	Female (n = 41)	Mean difference	t-value	P-value	Cohen's d
KOOS	89.6 ± 8.4	87.7 ± 9.1	1.9	0.88	0.382	0.22
IKDC	87.4 ± 6.5	85.8 ± 7.1	1.6	0.82	0.417	0.23
Lysholm	89.1 ± 6.3	87.7 ± 7.0	1.4	0.74	0.463	0.21

Activity levels, as assessed using the Tegner score, showed no significant difference between pre-injury and postoperative values. The mean pre-injury Tegner score was 6.3 ± 2.2, compared with 6.5 ± 1.8 at 12 months postoperatively (P = 0.544), suggesting a return to pre-injury activity levels. These findings are summarized in Table 3.

**Table 3:** Comparison of pre-injury and postoperative Tegner activity scores (n = 64) Values are mean ± SD where applicable. Paired comparisons were performed using a paired Student's t-test (t = 0.61, df = 63). CI: confidence interval, SD: standard deviation

Variable	Value
Tegner score, pre-injury	6.3 ± 2.2
Tegner score, 12 months postoperative	6.5 ± 1.8
Mean difference	0.14 ± 1.04
95% CI	−0.32 to 0.60
t-statistic	0.61
P-value	0.544
Cohen's d	0.13

Radiological evaluation demonstrated a mean SNQ value of 4.86 ± 1.55, consistent with the expected phase of early graft remodeling. SNQ values did not show significant associations with age, body mass index, or sex. These findings are summarized in Table 4.

**Table 4:** MRI graft maturation and isokinetic strength outcomes at six months (n = 64) SNQ (higher SNQ = greater graft signal intensity, reflecting less mature ligamentization); LSI (involved/uninvolved limb peak torque × 100; ≥90% considered adequate for return-to-sport clearance); and H/Q (≥60% considered adequate for knee stability). SD: standard deviation, MRI: magnetic resonance imaging, SNQ: signal-to-noise quotient, LSI: limb symmetry indices, H/Q: hamstring-to-quadriceps

Variable	Mean ± SD
SNQ	4.86 ± 1.55
Quadriceps LSI (%)	78.95 ± 5.90
Hamstring LSI (%)	83.59 ± 6.04
H/Q ratio (%)	59.59 ± 5.71

Isokinetic testing revealed progressive neuromuscular recovery, with a mean quadriceps LSI of 78.95% ± 5.90% and a mean hamstring LSI of 83.59% ± 6.04%. The H/Q ratio was 59.59% ± 5.71%, indicating relatively greater recovery of hamstring strength compared with quadriceps strength.

Correlation analysis demonstrated strong and statistically significant inverse relationships between SNQ and objective strength parameters. Specifically, SNQ was strongly correlated with quadriceps LSI (r = −0.87, P < 0.001), hamstring LSI (r = −0.95, P < 0.001), and the H/Q ratio (r = −0.95, P < 0.001), indicating that higher graft signal intensity was associated with reduced muscle strength symmetry.

In contrast, SNQ did not demonstrate significant correlations with patient-reported outcomes, including KOOS, IKDC, or Lysholm scores. Multivariable regression analysis confirmed that SNQ was a significant independent predictor of quadriceps strength recovery but not of subjective functional outcomes (Table 5).

**Table 5:** Multivariable linear regression: independent predictors of quadriceps LSI at six months Dependent variable: quadriceps LSI (%). Sex coded as male = 1, female = 0 (reference). Model statistics: F(4, 59) = 45.87, p < 0.001, R² = 0.757, adjusted R² = 0.74. β: unstandardized regression coefficient, SE: standard error, CI: confidence interval, BMI: body mass index, SNQ: signal-to-noise quotient, LSI: limb symmetry index

Predictor	β	SE	t-value	95% CI	P-value
SNQ	−2.85	0.29	−9.79	−3.42 to −2.28	<0.001
Age (years)	0.15	0.1	1.47	−0.05 to 0.35	0.14
BMI (kg/m²)	−0.30	0.28	−1.07	−0.85 to 0.25	0.28
Sex	1.2	1.38	0.87	−1.50 to 3.90	0.38

## Discussion

The most important finding of the present study is that reverse-passing ACL reconstruction combined with ALL reconstruction was associated with excellent early functional recovery and a low overall complication rate (1 of 64 patients requiring a minor surgical revision for hardware-related wound dehiscence), with no graft failures observed during follow-up. Furthermore, MRI-derived graft maturation demonstrated strong correlations with objective strength recovery, while showing no meaningful association with patient-reported outcomes.

The favorable outcomes observed in the study are consistent with the growing body of literature supporting the addition of lateral extra-articular procedures to ACL reconstruction. Recent papers looking at elite athletes, adolescent patients, and high-risk populations have consistently shown that ALL reconstruction and LET improve rotational stability and reduce graft failure rates [1,7-9].

Numerous studies have now demonstrated a significantly lower risk of graft rupture in elite athletes treated with combined ACL reconstruction and LET compared with isolated ACL reconstruction [1]. Furthermore, lower revision rates after LET augmentation have also been demonstrated in similar populations [7] together with superior clinical outcomes and lower residual instability [8]. The findings of our study are in agreement with these reports, as no episodes of recurrent instability or graft failure were observed during the early postoperative period.

The results of the STABILITY trial further support the role of lateral augmentation in high-risk patients. With younger age, high activity levels, generalized laxity, and high-grade pivot shift considered major predictors of graft failure, LET has been shown to significantly reduce the likelihood of reinjury [9]. These observations are particularly relevant to our cohort, which consisted predominantly of young active individuals who may be at elevated risk of recurrent instability.

Recent systematic reviews have confirmed the value of lateral extra-articular procedures in reducing graft failure, with combined reconstructions providing improved rotational stability and lower residual laxity without major increases in complications [2,6,10-11]. Additionally, similar papers have demonstrated favorable clinical and functional outcomes after combined ACL and ALL reconstruction, particularly in terms of knee stability and return to sport [20]. Taken together, these findings reinforce the concept that combined reconstruction may be particularly beneficial in active patients at high risk for residual rotational laxity.

The use of ALL reconstruction in this technique was based on the growing recognition of the anterolateral complex as an important secondary stabilizer of the knee. Biomechanical studies have shown that the ALL contributes to controlling internal tibial rotation, particularly during pivoting activities [3-4]. Both ALL reconstruction and LET provide similar biomechanical benefits, suggesting that both procedures may reduce the rotational load placed on the intra-articular ACL graft [4]. Likewise, the technical rationale for combining intra-articular and extra-articular procedures to improve rotational control and protect the graft has been highlighted as especially important during the early postoperative phase [5].

The indications for combined ACL and lateral extra-articular procedures continue to evolve. According to a recent international consensus statement [12] and multiple systematic reviews, major indications include young age, elite sports participation, generalized hyperlaxity, revision surgery, meniscal deficiency, and high-grade pivot shift. The patient population in the present study fits several of these criteria, further supporting the appropriateness of combined ACL and ALL reconstruction.

A particularly important finding was the strong inverse correlation between SNQ and objective strength recovery. Patients with higher SNQ values demonstrated lower quadriceps and hamstring limb symmetry indices, suggesting that greater graft signal intensity may reflect slower biological maturation and delayed neuromuscular recovery. This observation is clinically meaningful because it suggests that MRI may provide information beyond clinical examination alone when determining readiness for higher-level activity.

MRI-based evaluation of graft maturation has become increasingly important in ACL reconstruction research. Although previous studies have produced conflicting findings regarding the relationship between MRI signal intensity and clinical outcomes, the present results suggest that graft maturation may be more closely related to objective neuromuscular function than to subjective perception. This may explain why SNQ values were associated with strength recovery but not with patient-reported scores.

This lack of association between SNQ and patient-reported outcomes is clinically relevant. Subjective outcome measures such as KOOS, IKDC, and Lysholm are influenced by multiple factors beyond structural healing, including pain, confidence, fear of reinjury, and psychological readiness to return to sport. Therefore, patients may report excellent function despite incomplete biological graft maturation. This distinction is clinically important when considering return to sport, especially in elite athletes who may feel ready to return to high-level competition but may not yet have achieved full graft maturation.

The persistence of quadriceps deficits at six months is also consistent with previous literature. Quadriceps inhibition after ACL reconstruction remains a common challenge and may contribute to altered biomechanics, reduced performance, and increased reinjury risk. In contrast, hamstring recovery appeared relatively more advanced in this cohort, despite the use of hamstring tendon autografts. The observation that hamstring LSI (83.59%) exceeded quadriceps LSI (78.95%) at six months may appear counterintuitive and warrants clinical explanation. Several mechanisms likely account for this finding. First, quadriceps inhibition following ACL reconstruction is a well-documented neurophysiological phenomenon driven by pain, effusion, and altered afferent signaling from the reconstructed knee, which persists independently of the graft source and is not replicated on the donor side. Second, the hamstring musculature is more rapidly activated during closed-chain kinetic exercises, which dominate early rehabilitation, compared with the quadriceps, further favoring earlier hamstring recovery. Taken together, these factors explain the asymmetric recovery profile observed. They are consistent with the existing literature reporting preferential hamstring recovery over quadriceps symmetry in the early postoperative period following hamstring autograft ACL reconstruction.

The reverse-passing technique itself may also have contributed to the favorable findings observed in this study. Previous biomechanical investigations have shown that fixation methods influence tunnel motion, graft stability, and early healing. Many studies have demonstrated the importance of fixation biomechanics [14], while others have emphasized the rationale for combining intra-articular and extra-articular reconstruction strategies [5]. The reverse-passing method may provide improved graft tensioning and fixation characteristics, potentially supporting more favorable biological integration. Integration may also be improved by the half-tunnel technique on the tibia, which preserves important tibial bone stock. However, in the absence of a comparator arm, this hypothesis cannot be distinguished from the effects of ALL augmentation or broader single-surgeon technique standardization. It should be tested in future comparative studies.

Long-term data regarding combined reconstruction techniques are becoming increasingly available; recently published studies have demonstrated favorable long-term outcomes after ACL reconstruction using different surgical techniques at a minimum of 20 years of follow-up [21]. Another study identified persistent risk factors for graft failure in professional athletes, including younger age and return to pivoting sports, supporting the rationale for additional lateral stabilization in these populations [22]. More recently, promising five-year results from a randomized trial comparing combined ACL and ALL reconstruction using hamstring autografts with isolated bone-patellar tendon-bone ACL reconstruction [23-24] have been described, demonstrating lower graft failure rates and improved rotational stability in the combined reconstruction group, providing additional evidence in favor of lateral augmentation. Earlier reports describing clinical outcomes following combined ACL and ALL reconstruction similarly demonstrated favorable knee stability and functional recovery, supporting the durability of this combined approach across different surgical techniques.

An important additional finding of the study was that all patients returned to their baseline, preoperative Tegner activity scores and had all returned to sport by 12 months. This is consistent with recent literature and highlights the effectiveness of the technique. Although the majority of patients reported subjective readiness to return to sport at the six-month mark, return to sport was deliberately deferred until 12 months in accordance with our institutional protocol, as all patients were young and therefore considered at higher risk of early graft rerupture.

Regarding complications, the wound dehiscence observed in one patient (1.6%) occurred at the site of the knotless anchor system overlying Gerdy's tubercle. This region has limited soft tissue coverage, and superficial hardware prominence in this anatomical location may predispose to wound-healing complications, particularly in patients with a lean body habitus. We hypothesize that this complication was related to an idiosyncratic tissue reaction to the hardware rather than a systematic technical failure, given the absence of similar events in the remaining cohort despite identical anchor placement. To minimize this risk, surgeons should ensure adequate soft tissue interposition between the anchor and the skin. They may consider subperiosteal anchor burial or use of an absorbable fixation system in patients with thin subcutaneous tissue over the proximal tibia.

The findings of the study have several important clinical implications. First, they support the use of combined ACL and ALL reconstruction in high-risk populations to improve rotational stability and potentially reduce graft failure. Second, they demonstrate that MRI-derived graft maturation may provide useful information regarding neuromuscular recovery. Finally, they reinforce the importance of combining imaging, strength testing, and patient-reported measures when evaluating readiness to return to sport.

Despite its strengths, this study has several limitations. The relatively short follow-up period limits the assessment of long-term graft survival and return-to-sport durability. The absence of a control group precludes direct comparison with isolated ACL reconstruction or other lateral augmentation techniques. This means that the favorable outcomes observed cannot be attributed specifically to the reverse-passing technique, but rather to ALL augmentation more generally or to the standardized care provided by a single experienced surgeon. Finally, the single-surgeon design may limit the generalizability of the findings.

Future studies should investigate long-term graft survival, rate of return to sport, and comparative outcomes between reverse-passing ACL and ALL reconstruction and more conventional techniques.

## Conclusions

In this single-arm preliminary series, reverse-passing ACL reconstruction with concomitant ALL reconstruction was associated with excellent functional recovery at six months, a high rate of return to pre-injury activity levels and sport participation by 12 months, and a low complication rate. MRI-based graft maturation correlated strongly with objective neuromuscular recovery but not with patient-reported functional outcomes, suggesting that these two dimensions of recovery are partially independent. These findings should be interpreted within the constraints of a retrospective single-arm design without a comparator group, which precludes attribution of the observed outcomes to the reverse-passing technique specifically, as opposed to the concomitant ALL reconstruction, the rehabilitation protocol, or surgeon experience. Comparative prospective studies are required to determine whether the reverse-passing technique confers any specific advantage over conventional ACL + ALL techniques.
